# Baseline inflammatory and metabolic indicators associated with early PD-1 inhibitor resistance in advanced cervical cancer: a retrospective cohort study

**DOI:** 10.3389/fmed.2026.1779898

**Published:** 2026-03-16

**Authors:** Zhao Xiaoyan, Liu Xiaojuan, Mo Shijiao, Zhou Xiaofan

**Affiliations:** 1 Department of Obstetrics and Gynecology, The First Affiliated Hospital of Hebei North University, Zhangjiakou, Hebei, China; 2 Emergency Department, Shijiazhuang Maternal and Child Health Hospital, Shijiazhuang, Hebei, China; 3 Department of Obstetrics, Shijiazhuang Maternal and Child Health Care Hospital, Shijiazhuang, Hebei, China

**Keywords:** cervical cancer, immune resistance, lactate dehydrogenase (LDH), neutrophil-to-lymphocyte ratio (NLR), PD-1 inhibitor, predictive model, systemic inflammation

## Abstract

**Background:**

Despite the clinical success of PD-1/PD-L1 inhibitors, resistance remains a major barrier to durable responses in advanced cervical cancer. Identifying readily available biomarkers predictive of immunotherapy resistance may improve treatment selection and patient outcomes.

**Methods:**

This single-center retrospective cohort study included 140 patients with histologically confirmed advanced or recurrent cervical cancer who received at least two cycles of PD-1 inhibitor therapy. Clinical characteristics, laboratory indices—including neutrophil-to-lymphocyte ratio (NLR) and lactate dehydrogenase (LDH)—and prior treatment history were analyzed. Resistance was defined as confirmed progressive disease (iCPD) according to iRECIST criteria. Logistic regression and receiver operating characteristic (ROC) analyses were performed to identify independent predictors and evaluate model performance.

**Results:**

Of 140 patients, 68 (48.6%) developed PD-1 inhibitor resistance. Resistant cases exhibited significantly higher baseline NLR (4.09 ± 1.64 vs. 3.02 ± 1.28, *p* < 0.001), elevated LDH (264.4 ± 88.9 U/L vs. 216.8 ± 69.4 U/L, *p* = 0.003), and more frequent prior chemotherapy (67.6% vs. 47.2%, *p* = 0.018). In multivariable logistic regression analysis, baseline NLR (OR = 1.62, 95% CI 1.21–2.17, *p* = 0.002), LDH (OR = 1.05 per 10 U/L increase, 95% CI 1.01–1.09, *p* = 0.012), and prior chemotherapy (OR = 2.08, 95% CI 1.01–4.28, *p* = 0.047) were independently associated with early PD-1 inhibitor resistance. The combined model incorporating NLR, LDH, PD-L1 expression, and prior chemotherapy achieved an AUC of 0.842 (95% CI 0.773–0.911), outperforming individual parameters (*p* < 0.05). Exploratory analysis showed that PD-1–based combination regimens achieved a disease control rate of 72.2% in previously resistant patients.

**Conclusion:**

Systemic inflammatory and metabolic markers, together with clinical treatment history, can effectively predict PD-1 inhibitor resistance in advanced cervical cancer.

## Introduction

Cervical cancer remains one of the most prevalent malignancies among women worldwide, representing a major public health challenge despite advances in prevention and early detection (1–3). According to the latest global cancer statistics, approximately 600,000 new cases and 340,000 deaths occur each year, with a disproportionate burden in low- and middle-income countries (4). Although HPV vaccination and routine screening have markedly reduced the incidence of early-stage disease, the prognosis for advanced or recurrent cervical cancer remains poor (3, 5). Standard first-line chemotherapy, with or without anti-angiogenic agents such as bevacizumab, provides only modest benefit, with a median overall survival of approximately 17 months (6, 7). These figures underscore the urgent need for more effective systemic therapies and reliable biomarkers to guide treatment decisions in this population.

In recent years, immune checkpoint inhibitors targeting the programmed cell death 1 (PD-1) and programmed death ligand 1 (PD-L1) axis have significantly reshaped the therapeutic landscape of advanced cervical cancer (8–10). In the phase II KEYNOTE-158 trial, pembrolizumab monotherapy achieved an objective response rate (ORR) of 14.3% in PD-L1–positive recurrent or metastatic cervical cancer (11). Despite these advances, most patients eventually experience primary or acquired resistance, and only a small fraction derive durable benefit (9).

Although PD-1/PD-L1 blockade has transformed cancer therapy, immune resistance remains a major challenge (12, 13). It results from complex interactions between tumor cells and an immunosuppressive tumor microenvironment (14–16). Tumor-intrinsic alterations—such as loss of antigen presentation, defective interferon signaling, and oncogenic pathway activation—can impair cytotoxic T-cell function, while the microenvironment is often enriched in regulatory T cells, myeloid-derived suppressor cells, and inhibitory cytokines (IL-6, TGF-*β*) (12, 17, 18). In addition, systemic inflammation and metabolic dysregulation play critical roles (19–22). An elevated neutrophil-to-lymphocyte ratio (NLR) reflects chronic inflammation and weakened immune surveillance, whereas increased lactate dehydrogenase (LDH) indicates enhanced glycolysis and hypoxia, promoting immune escape (23–27). These accessible biomarkers mirror the host–tumor immune balance, yet their predictive value in cervical cancer treated with PD-1 inhibitors remains largely unexplored.

Previous studies have largely focused on tumor-intrinsic factors such as PD-L1 expression or tumor mutational burden, with inconsistent predictive performance (9, 28–30). Few efforts have integrated easily accessible clinical and inflammatory markers into a multivariable predictive framework, and data on strategies to overcome PD-1 resistance in cervical cancer remain scarce. Therefore, this study aimed to identify readily available clinical and inflammatory factors associated with early PD-1 inhibitor resistance in advanced cervical cancer and to develop a combined model for risk stratification of early treatment non-benefit. In addition, an exploratory analysis was conducted to evaluate the short-term efficacy of PD-1–based combination therapies in patients with initial resistance.

## Methods

### Study design and population

This single-center, retrospective cohort study was conducted at the Department of Oncology, The First Affiliated Hospital of Hebei North University (Zhangjiakou, China) between January 2020 and June 2024. A total of 140 female patients with histologically confirmed advanced or recurrent cervical cancer who received PD-1 inhibitor–based immunotherapy were included in the analysis.

All participants had measurable disease, which was evaluated according to the immune Response Evaluation Criteria in Solid Tumors (iRECIST) and completed at least two cycles of PD-1 inhibitor therapy, followed by radiologic assessment at 12 ± 2 weeks. Based on treatment response, patients were classified into two groups: the PD-1–resistant group (*n* = 68), defined as those with confirmed progressive disease (iCPD), and the non-resistant group (*n* = 72), which included patients who achieved complete response, partial response, or stable disease (CR/PR/SD).

This study was conducted in accordance with the ethical standards of the Declaration of Helsinki and approved by the Ethics Committee of The First Affiliated Hospital of Hebei North University.

### Inclusion and exclusion criteria

Patients were eligible for inclusion if they met all of the following conditions: (1) Histologically confirmed cervical cancer (including squamous cell carcinoma, adenocarcinoma, or adenosquamous carcinoma) diagnosed according to the World Health Organization (WHO) classification of tumors of the uterine cervix; (2) Advanced, recurrent, or metastatic disease not amenable to curative surgery or radiotherapy; (3) Received at least two consecutive cycles of PD-1 inhibitor–based therapy (pembrolizumab, camrelizumab, toripalimab, or sintilimab) as monotherapy or in combination with chemotherapy/anti-angiogenic agents; (4) Completed radiologic response assessment at 12 ± 2 weeks after treatment initiation according to iRECIST criteria; (5) Availability of complete clinical, pathological, and laboratory data, including hematologic and biochemical parameters (e.g., neutrophil count, lymphocyte count, LDH, CEA, CA125, albumin, hemoglobin).

Patients meeting any of the following were excluded: (1) History or coexistence of other malignant tumors; (2) Presence of autoimmune diseases, chronic inflammatory disorders, or active infections (including hepatitis or HIV); (3) Receipt of systemic corticosteroids or immunosuppressive therapy within four weeks prior to PD-1 treatment; (4) Incomplete follow-up or imaging data, preventing accurate response evaluation; (5) Poor general condition (ECOG performance status > 2) or severe organ dysfunction precluding treatment.

### Treatment regimens

All patients received anti–PD-1 inhibitor therapy as either monotherapy or in combination with chemotherapy and/or anti-angiogenic agents. The PD-1 inhibitors used in this study included pembrolizumab (200 mg, q3w), camrelizumab (200 mg, q3w), toripalimab (240 mg, q3w), and sintilimab (200 mg, q3w), administered intravenously according to standard dosing schedules. Combination regimens primarily consisted of PD-1 inhibitors plus platinum-based chemotherapy (paclitaxel–cisplatin or paclitaxel–carboplatin), or PD-1 inhibitors plus anti–VEGF therapy (bevacizumab 15 mg/kg, q3w). Treatment continued until radiologic disease progression, unacceptable toxicity, or patient withdrawal. Prior to PD-1 inhibitor initiation, detailed records of previous treatments were collected, including prior chemotherapy, radiotherapy, or surgical intervention. Most patients had received at least one line of systemic chemotherapy before immunotherapy, and those with prior radiotherapy were required to have completed it at least 4 weeks before enrollment.

### Data collection and variables

#### Data sources

All clinical information was retrieved from the electronic medical record system, picture archiving and communication system (PACS), and laboratory information system. Data extraction and verification were performed independently by two investigators to ensure completeness and accuracy. HPV infection status, determined by routine clinical testing (HPV DNA testing or pathology records), was collected when available.

#### Clinical variables

The following baseline clinical parameters were collected: age, body mass index (BMI), FIGO stage, histological subtype, PD-L1 expression status, and treatment history (including prior chemotherapy, radiotherapy, or surgical intervention). PD-L1 expression was assessed by immunohistochemistry using the tumor proportion score (TPS), defined as the percentage of viable tumor cells showing membranous staining. PD-L1 positivity was defined as TPS ≥ 1%.

#### Laboratory indicators

Laboratory data included neutrophil-to-lymphocyte ratio (NLR), lactate dehydrogenase (LDH), hemoglobin (Hb), albumin (Alb), carcinoembryonic antigen (CEA), and carbohydrate antigen 125 (CA125). NLR was calculated as the absolute neutrophil count divided by the absolute lymphocyte count. All measurements were obtained within 7 days before the initiation of PD-1 inhibitor therapy.

#### Outcome variable

The treatment outcome was defined according to iRECIST criteria. Patients with confirmed progressive disease (iCPD) were classified as PD-1–resistant, whereas those achieving complete response (CR), partial response (PR), or stable disease (SD) were categorized as non-resistant.

#### Data quality control

Data quality control was performed before analysis. Outliers greater than three standard deviations from the mean were carefully rechecked and excluded if confirmed to be biologically implausible. Patients with missing key variables (e.g., NLR or LDH) or incomplete imaging evaluations were excluded from the final analysis dataset.

### Response evaluation

#### Evaluation criteria

Tumor response was evaluated according to the immune Response Evaluation Criteria in Solid Tumors (iRECIST), which are specifically designed for assessing treatment outcomes during immunotherapy. Baseline imaging (CT or MRI) was performed within 4 weeks prior to initiation of PD-1 inhibitor therapy, and the first follow-up imaging assessment was conducted at approximately 12 weeks (±2 weeks) after treatment initiation, consistent with routine clinical practice.

Early clinical deterioration without radiologic confirmation was not classified as disease progression in accordance with iRECIST recommendations. Progressive disease was defined as immune confirmed progressive disease (iCPD) based on radiologic evidence. In this cohort, no cases of immune unconfirmed progressive disease (iUPD) requiring subsequent confirmation were identified.

#### Definition of response categories

According to iRECIST, tumor response was categorized as complete response (iCR), defined as the disappearance of all target and non-target lesions; partial response (iPR), defined as at least a 30% decrease in the sum of target lesion diameters; stable disease (iSD), indicating insufficient shrinkage to meet the criteria for iPR and insufficient increase to qualify as progressive disease; progressive disease (iPD), defined as at least a 20% increase in the sum of target lesion diameters or the appearance of new lesions; and confirmed progressive disease (iCPD), which required progression to be verified by repeat imaging after 4–8 weeks to exclude pseudoprogression.

#### Definition of resistance

For the purpose of this study, PD-1 resistance was defined as iCPD confirmed on repeat imaging. Patients who achieved iCR, iPR, or iSD were classified as non-resistant. All radiologic assessments were independently reviewed by two experienced radiologists blinded to the clinical and laboratory data, and any discrepancies were resolved by consensus.

### Statistical analysis

All statistical analyses were performed using SPSS software (version 26.0; IBM Corp., Armonk, NY, United States) and R software (version 4.3.1; R Foundation for Statistical Computing, Vienna, Austria). Data distribution was examined using the Shapiro–Wilk test to determine normality. Continuous variables were expressed as mean ± standard deviation (SD) for normally distributed data or as median (interquartile range, IQR) for non-normal data. Comparisons between groups were conducted using the independent-samples t-test or the Mann–Whitney U test, as appropriate. Categorical variables were presented as counts and percentages and compared using the chi-square test or Fisher’s exact test.

Univariate logistic regression was used to identify potential predictors of PD-1 inhibitor resistance. Variables with *p* < 0.10 in univariate analysis or with clear clinical relevance were subsequently entered into a multivariate logistic regression model using the Enter method to determine independent risk factors. Results were reported as odds ratios (ORs) with 95% confidence intervals (CIs) and corresponding *p* values. Variables with *p* < 0.10 in univariate analysis were entered into the multivariable logistic regression model using the Enter method. In addition, PD-L1 expression was retained irrespective of statistical significance because of its established clinical relevance in immunotherapy response assessment. This hypothesis-driven approach was chosen to ensure adjustment for clinically meaningful covariates and to minimize potential confounding.

Model discrimination was assessed using receiver operating characteristic (ROC) curve analysis, and area under the curve (AUC) values were compared by the DeLong test. The optimal cut-off point for each variable was determined according to the Youden index (sensitivity + specificity − 1).

Subgroup analyses were conducted according to treatment modality, PD-L1 expression, and disease stage. Sensitivity analyses tested the stability of the model using alternative cut-offs for NLR and LDH, as well as exclusion of cases with missing or extreme values. An exploratory analysis was performed among PD-1–resistant patients who subsequently received PD-1–based combination therapy (PD-1 + chemotherapy or PD-1 + anti-VEGF). Short-term outcomes were compared using the disease control rate (DCR), evaluated by the χ^2^ test. A two-tailed *p* value < 0.05 was considered statistically significant for all analyses.

## Results

### Baseline characteristics

A total of 140 patients with advanced cervical cancer were included in the analysis, of whom 68 (48.6%) were classified as PD-1–resistant and 72 (51.4%) as non-resistant (CR/PR/SD) based on the iRECIST criteria at the 12 ± 2-week evaluation (Table 1). The mean age of the study population was 54.12 ± 9.84 years, and the mean BMI was 23.68 ± 3.42 kg/m^2^, with no significant differences between the two groups (both *p* > 0.05). The majority of patients were diagnosed at FIGO stage III (55.7%), and 78.6% had squamous cell carcinoma histology. PD-L1 expression (≥1%) was observed in 59.3% of all patients and was slightly more frequent in the non-resistant group (66.7% vs. 51.5%, *p* = 0.072). Regarding prior treatment, 67.6% of resistant patients had received previous chemotherapy compared with 47.2% of non-resistant patients (*p* = 0.018), whereas prior radiotherapy was comparable between groups (69.1% vs. 62.5%, *p* = 0.419). At baseline, resistant patients showed significantly higher levels of systemic inflammation and metabolic activity. The mean NLR was markedly elevated in the resistant group (4.09 ± 1.64 vs. 3.02 ± 1.28, *p* < 0.001), and the mean LDH level was also significantly higher (264.37 ± 88.92 U/L vs. 216.75 ± 69.43 U/L, *p* = 0.003). Other biochemical parameters, including hemoglobin (118.06 ± 14.02 g/L vs. 120.69 ± 12.46 g/L, *p* = 0.298), albumin (39.13 ± 4.25 g/L vs. 40.35 ± 3.94 g/L, *p* = 0.186), and tumor markers such as CEA and CA125, did not differ significantly between groups (all *p* > 0.05). HPV positivity was observed in 88.6% of patients overall and did not differ significantly between the resistant and non-resistant groups (86.8% vs. 90.3%, *p* = 0.421). The distribution of treatment regimens did not differ significantly between resistant and non-resistant groups (Supplementary Table S1).

**Table 1:** Baseline characteristics of the study population stratified by PD-1 inhibitor resistance status.

Variable	Total (*n* = 140)	Resistant (PD) *n* = 68	Non-resistant (CR/PR/SD) *n* = 72	*p* value
Age (years), mean ± SD	54.12 ± 9.84	55.47 ± 10.21	52.86 ± 9.37	0.147
BMI (kg/m^2^), mean ± SD	23.68 ± 3.42	23.51 ± 3.55	23.83 ± 3.29	0.543
FIGO stage, *n* (%)				0.317
III	78 (55.71)	41 (60.29)	37 (51.39)	
IV	62 (44.29)	27 (39.71)	35 (48.61)	
Histologic type, *n* (%)				0.476
Squamous cell carcinoma	110 (78.57)	55 (80.88)	55 (76.39)	
Adenocarcinoma	30 (21.43)	13 (19.12)	17 (23.61)	
HPV status, *n* (%)				0.421
HPV positive	124 (88.57)	124 (88.57)	124 (88.57)	
HPV negative	16 (11.43)	9 (13.24)	7 (9.72)	
PD-L1 expression (≥1%), *n* (%)	83 (59.29)	35 (51.47)	48 (66.67)	0.072
Prior chemotherapy, *n* (%)	80 (57.14)	46 (67.65)	34 (47.22)	0.018 *
Prior radiotherapy, *n* (%)	92 (65.71)	47 (69.12)	45 (62.50)	0.419
Baseline NLR, mean ± SD	3.53 ± 1.52	4.09 ± 1.64	3.02 ± 1.28	< 0.001 *
Baseline LDH (U/L), mean ± SD	239.41 ± 81.67	264.37 ± 88.92	216.75 ± 69.43	0.003 *
Hemoglobin (g/L), mean ± SD	119.42 ± 13.27	118.06 ± 14.02	120.69 ± 12.46	0.298
Albumin (g/L), mean ± SD	39.76 ± 4.12	39.13 ± 4.25	40.35 ± 3.94	0.186
CEA (ng/mL), median (IQR)	2.91 (1.72–5.48)	3.25 (1.88–5.96)	2.58 (1.61–4.97)	0.229
CA125 (U/mL), median (IQR)	23.87 (12.14–45.63)	25.94 (13.11–47.82)	22.05 (11.47–43.56)	0.331

*p < 0.05 was considered statistically significant. Comparisons were performed using the χ^2^ test for categorical variables.

### Logistic regression analysis of predictors for PD-1 inhibitor resistance

In the univariate logistic regression analysis, several baseline factors were associated with PD-1 inhibitor resistance, including higher NLR (OR = 1.74, 95% CI: 1.33–2.29, *p* < 0.001), elevated LDH (OR = 1.06 per 10 U/L increase, 95% CI: 1.02–1.10, *p* = 0.004), prior chemotherapy exposure (OR = 2.36, 95% CI: 1.21–4.63, *p* = 0.012), and lower PD-L1 expression (*p* = 0.061). Other demographic and biochemical variables showed no significant associations (all *p* > 0.10). Variables with *p* < 0.10 in univariate analysis were entered into the multivariate logistic regression model. After adjustment for confounders, NLR (OR = 1.62, 95% CI: 1.21–2.17, *p* = 0.002), LDH (OR = 1.05 per 10 U/L, 95% CI: 1.01–1.09, *p* = 0.012), and prior chemotherapy (OR = 2.08, 95% CI: 1.01–4.28, *p* = 0.047) remained independent predictors of PD-1 inhibitor resistance. PD-L1 expression showed a borderline protective trend (*OR* = 0.58, *p* = 0.121) but did not reach statistical significance (Table 2).

**Table 2:** Univariate and multivariate logistic regression analyses of predictors for PD-1 inhibitor resistance.

Variable	Univariate OR (95% CI)	*p* value	Multivariate OR (95% CI)	*p* value
Age (years)	1.02 (0.98–1.06)	0.276	—	—
BMI (kg/m^2^)	0.97 (0.88–1.08)	0.576	—	—
FIGO stage (IV vs III)	0.74 (0.38–1.43)	0.374	—	—
Histology (adenocarcinoma vs squamous)	0.83 (0.37–1.87)	0.656	—	—
PD-L1 expression (≥1% vs <1%)	0.54 (0.28–1.03)	0.061	0.58 (0.29–1.16)	0.121
Prior chemotherapy (yes vs no)	2.36 (1.21–4.63)	0.012*	2.08 (1.01–4.28)	0.047*
Prior radiotherapy (yes vs no)	1.34 (0.68–2.63)	0.405	—	—
Baseline NLR (per 1-unit increase)	1.74 (1.33–2.29)	<0.001*	1.62 (1.21–2.17)	0.002*
Baseline LDH (per 10 U/L increase)	1.06 (1.02–1.10)	0.004*	1.05 (1.01–1.09)	0.012*
Hemoglobin (per 1 g/L increase)	0.98 (0.94–1.01)	0.153	—	—
Albumin (per 1 g/L increase)	0.94 (0.86–1.02)	0.143	—	—
CEA (log-transformed)	1.21 (0.81–1.81)	0.353	—	—
CA125 (log-transformed)	1.09 (0.91–1.31)	0.356	—	—

*p < 0.05 was considered statistically significant. —, variable not retained in the multivariate model (entry criterion *p* < 0.10).

### ROC curve analysis

**Figure 1:**
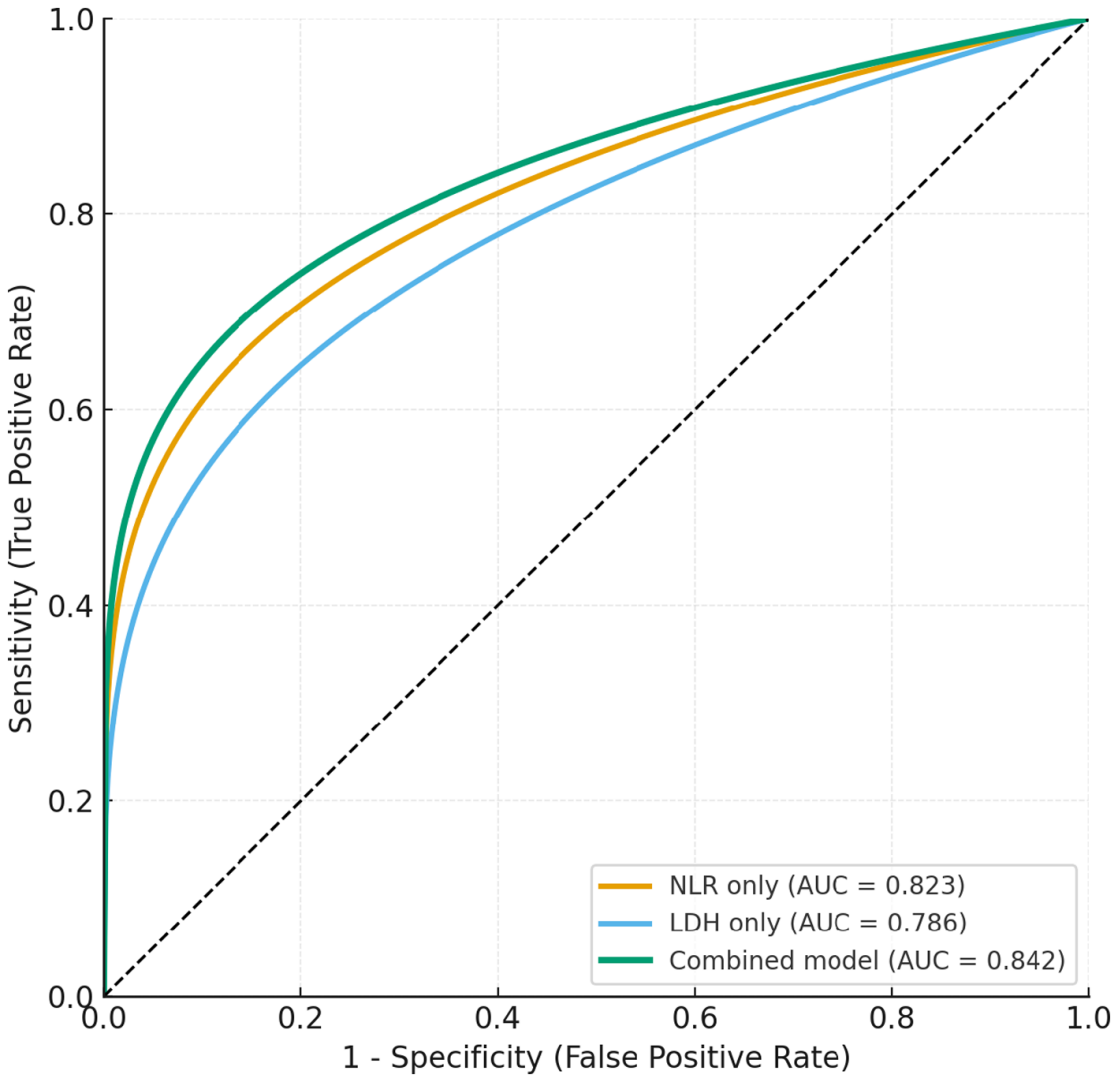
Receiver operating characteristic (ROC) curves comparing the discriminative performance of single predictors (NLR, LDH) and the combined multivariable model (NLR + LDH + PD-L1 + prior chemotherapy) for identifying PD-1 inhibitor resistance. The combined model demonstrated superior discrimination (AUC = 0.842) compared with either single-parameter model (DeLong *p* < 0.05). Shaded areas indicate 95% confidence intervals.

The discriminative performance of individual and combined predictive models for PD-1 inhibitor resistance was evaluated using receiver operating characteristic (ROC) curve analysis. As shown in Figure 1, both baseline NLR and LDH alone demonstrated good diagnostic ability, while the combined model integrating NLR, LDH, PD-L1 expression, and prior chemotherapy history achieved the best overall discrimination. The AUC of the NLR model was 0.823 (95% CI 0.747–0.898) and that of LDH was 0.786 (95% CI 0.703–0.868). The combined model reached an AUC of 0.842 (95% CI 0.773–0.911), significantly higher than either single-variable model (*p* < 0.05, DeLong test). At the optimal cut-off derived from the Youden index, the combined model yielded a sensitivity of 80.6%, specificity of 76.4%, and Youden index of 0.57, indicating good discriminative performance between resistant and non-resistant cases (Table 3).

**Table 3:** Diagnostic performance of different predictive models for PD-1 inhibitor resistance.

Model	AUC (95% CI)	Optimal Cut-off	Sensitivity (%)	Specificity (%)	Youden index	*p* value (vs combined)
NLR only	0.823 (0.747–0.898)	3.45	77.9	74.3	0.52	0.041*
LDH only	0.786 (0.703–0.868)	238.5 U/L	73.5	72.2	0.46	0.017*
PD-L1 (≥1%) only	0.612 (0.513–0.712)	—	61.1	57.6	0.19	<0.001*
Prior chemotherapy only	0.661 (0.567–0.755)	—	67.6	58.3	0.26	0.006*
Combined (NLR + LDH + PD-L1 + Chemo)	0.842 (0.773–0.911)	0.50 (predicted probability)	80.6	76.4	0.57	—

*p < 0.05 vs combined model by DeLong test. AUC, area under the ROC curve.

### Sensitivity analyses and subgroup analyses

Sensitivity tests (Supplementary Table S2) confirmed the robustness of the predictive model. Using alternative NLR (3.0–3.8) or LDH (230–250 U/L) thresholds, excluding cases with missing data or extreme values, and performing leave-one-out validation all produced similar AUC values (0.837–0.846), indicating excellent model stability. Subgroup results (Supplementary Table S3) showed consistent discrimination across treatment regimens (PD-1 monotherapy vs. combination therapy), PD-L1 expression levels, and FIGO stages (III–IVa vs. IVb). No significant AUC differences were observed between any subgroups (all *p* > 0.05), confirming the model’s applicability across clinical contexts.

### Reversal strategies

To preliminarily evaluate potential strategies for overcoming PD-1 inhibitor resistance, an exploratory analysis was conducted among 18 resistant patients who subsequently received PD-1–based combination therapy after initial monotherapy failure. Two major combination patterns were identified: PD-1 + chemotherapy (*n* = 10) and PD-1 + anti-VEGF therapy (*n* = 8). As summarized in Table 4, both regimens achieved encouraging short-term disease control. The overall disease control rate (DCR) among these previously resistant patients was 72.2%, with no significant difference between the two strategies (*p* = 0.641). Specifically, the PD-1 + chemotherapy group showed a DCR of 70.0% (7/10), while the PD-1 + anti-VEGF group achieved 75.0% (6/8).

**Table 4:** Disease control outcomes among resistant patients receiving PD-1–based combination therapy.

Combination strategy	n	CR + PR	SD	PD	DCR [%] (CR + PR + SD)	χ^2^ / *p* value
PD-1 + Chemotherapy	10	3 (30.0)	4 (40.0)	3 (30.0)	70.0%	
PD-1 + Anti-VEGF therapy	8	2 (25.0)	4 (50.0)	2 (25.0)	75.0%	
Total	18	5 (27.8)	8 (44.4)	5 (27.8)	72.2%	χ^2^ = 0.219 / *p* = 0.641

CR, complete response; PR, partial response; SD, stable disease; PD, progressive disease; DCR, disease control rate = CR + PR + SD.

## Discussion

In this retrospective cohort study of patients with advanced or recurrent cervical cancer, we identified several clinically accessible biomarkers—including the NLR, LDH, prior chemotherapy history, and PD-L1 expression—as independent predictors of early resistance to PD-1 inhibitor therapy. The combined multivariable model incorporating these factors demonstrated good discriminative performance, with an area under the ROC curve (AUC) of approximately 0.84, indicating favorable predictive capability. Furthermore, an exploratory analysis suggested that PD-1–based combination regimens (with chemotherapy or anti-VEGF therapy) may partially mitigate immune resistance in selected patients. Collectively, these findings provide insight into risk stratification and may support more individualized optimization of immunotherapy strategies for advanced cervical cancer.

It is important to distinguish predictive from prognostic interpretations in immunotherapy research. In the present study, the identified markers should be interpreted as predictors of early iRECIST-defined treatment resistance, reflecting lack of early therapeutic benefit from PD-1 inhibitor therapy rather than indicators of survival outcomes. Because survival endpoints were not included in the original study design, no conclusions regarding PFS or OS can be drawn. Prospective studies with standardized follow-up are warranted to determine whether these baseline indicators also possess prognostic value.

Although PD-1/PD-L1 inhibitors have changed the treatment landscape of advanced cervical cancer, reliable predictors of therapeutic response remain lacking (31, 32). Most studies have focused on tumor-intrinsic factors such as PD-L1 expression, while host-related systemic indicators have been largely overlooked (33). Growing evidence from other cancers suggests that inflammation and metabolic dysregulation are closely linked to immune resistance, yet their combined predictive value in cervical cancer has not been systematically evaluated (34–36).

Previous studies on immunotherapy in cervical cancer have primarily focused on tumor-intrinsic biomarkers, such as PD-L1 expression, tumor mutational burden (TMB), and microsatellite instability (MSI), which reflect tumor immunogenicity but fail to capture the broader host immune context (37–41). Our findings extend this perspective by demonstrating that systemic inflammatory and metabolic markers—specifically NLR and LDH—also play pivotal roles in predicting resistance to PD-1 inhibitors. Similar associations between elevated NLR or LDH and poor immunotherapy outcomes have been reported in non–small cell lung cancer and melanoma, supporting their general relevance across tumor types (42–49). However, integrative models combining inflammatory, metabolic, and clinical variables have rarely been investigated in cervical cancer, highlighting the potential translational relevance of our approach.

The observed associations between NLR, LDH, and PD-1 inhibitor resistance are biologically plausible and reflect the interplay between systemic immunity and tumor metabolism. An elevated NLR indicates a shift toward a pro-tumor inflammatory state, where neutrophils promote tumor progression through cytokine release and suppression of T-cell activation, while reduced lymphocyte counts signal immune exhaustion and impaired surveillance (50–52). Similarly, an increase in LDH reflects enhanced glycolytic activity and lactate accumulation within the tumor microenvironment, leading to acidic conditions that suppress cytotoxic T-cell function and promote immune evasion (53, 54). In addition, a history of prior chemotherapy may further compromise immune competence by inducing T-cell apoptosis and reducing memory cell reserves (55, 56). Clinically, these factors provide valuable, readily measurable indicators for early identification of patients less likely to benefit from PD-1–based immunotherapy and may inform the design of personalized treatment strategies.

The proposed model may provide a practical framework for pretreatment risk stratification, potentially helping to identify patients more likely to benefit from PD-1–based therapy. Our exploratory findings further suggest that combination approaches, particularly PD-1 inhibitors combined with chemotherapy or anti-angiogenic agents, may represent promising strategies to mitigate treatment resistance. Prospective validation and mechanistic investigations are warranted to confirm these observations.

Several limitations of this study should be acknowledged. First, as a single-center retrospective cohort study with a relatively limited sample size, potential selection bias cannot be excluded. Second, although the retrospective cohort design allowed assessment of temporal associations between baseline biomarkers and early treatment resistance, causal inference remains limited. Third, survival outcomes such as progression-free survival (PFS) and overall survival (OS) were not analyzed because the study was specifically designed to evaluate early treatment resistance at the first iRECIST assessment, and survival follow-up was not uniformly available in this real-world cohort. Therefore, the identified markers should be interpreted as response-associated indicators rather than definitive prognostic biomarkers. Fourth, heterogeneity in PD-L1 assessment and the lack of direct characterization of the tumor immune microenvironment may have influenced the precision of biomarker interpretation. Future studies should validate these findings in large, multicenter prospective cohorts with standardized follow-up and integrate radiomic and multi-omics approaches, including immunogenomic and metabolomic profiling, to further improve predictive accuracy. In addition, mechanistic investigations exploring pathways of immune resistance and potential reversal strategies may enhance the clinical applicability of these observations.

## Conclusion

In summary, systemic inflammatory and metabolic markers, combined with clinical treatment history, may serve as useful indicators of PD-1 inhibitor resistance in advanced cervical cancer. These findings highlight the important role of host–tumor interactions in shaping immunotherapy outcomes and may contribute to improved risk stratification and the future development of personalized therapeutic strategies.

## Data Availability

The original contributions presented in the study are included in the article/Supplementary material, further inquiries can be directed to the corresponding author.
