# Supplementary material for: Baseline inflammatory and metabolic indicators associated with early PD-1 inhibitor resistance in advanced cervical cancer: a retrospective cohort study
Source: Front Med (Lausanne). 2026 Mar 16;13:1779898. doi: 10.3389/fmed.2026.1779898 (PMC13033690; doi:10.3389/fmed.2026.1779898)
Supplement: Supplementary file 1 [file Data_sheet_1.pdf]

Analysis Code (R) - PD-1 Resistance in Advanced Cervical Cancer

Dataset: PD1_Cervical_Cancer_Raw_Data.csv | Outputs: Table 1-4 workflow, ROC + DeLong, sensitivity/subgroups

```
# ============================================================
# Analysis Script (R)
# Study: Predictive Value of Systemic Inflammatory and Metabolic Markers
#         for PD-1 Inhibitor Resistance in Advanced Cervical Cancer
# Design: Single-center cross-sectional study
# Dataset: PD1_Cervical_Cancer_Raw_Data.csv
# ============================================================

# ---- 0) Setup ----
# Install packages if needed:
# install.packages(c("tidyverse","tableone","pROC","broom","rms","ResourceSelection","DescTools"))

library(tidyverse)
library(tableone)
library(pROC)
library(broom)
library(ResourceSelection)  # Hosmer-Lemeshow
library(DescTools)          # YoudenIndex

set.seed(123)

# ---- 1) Load data ----
dat <- read.csv("PD1_Cervical_Cancer_Raw_Data.csv", stringsAsFactors = FALSE)

# Recode for clarity
dat <- dat %>%
  mutate(
    PD1_Resistance = as.integer(PD1_Resistance),
    FIGO_Stage = factor(FIGO_Stage, levels = c("III","IV")),
    Histology = factor(Histology),
    PDL1_Positive = factor(PDL1_Positive, levels = c(0,1), labels = c("Negative","Positive")),
    Prior_Chemotherapy = factor(Prior_Chemotherapy, levels = c(0,1), labels = c("No","Yes")),
    Prior_Radiotherapy = factor(Prior_Radiotherapy, levels = c(0,1), labels = c("No","Yes"))
  )

# Define groups
dat$Group <- factor(dat$PD1_Resistance, levels = c(0,1),
                    labels = c("Non-resistant (CR/PR/SD)","Resistant (iCPD)"))

# ---- 2) Baseline characteristics (Table 1) ----
vars_cont <- c("Age","BMI","NLR","LDH_U_L","Hemoglobin_g_L","Albumin_g_L","CEA_ng_mL","CA125_U_mL")
vars_cat  <- c("FIGO_Stage","Histology","PDL1_Positive","Prior_Chemotherapy","Prior_Radiotherapy")

# For skewed markers, you may prefer median(IQR): here we keep flexibility
# Identify non-normal variables (optional)
# shapiro.test(dat$CEA_ng_mL)  # caution: large n may flag minor deviation

tab1 <- CreateTableOne(vars = c(vars_cont, vars_cat),
                       strata = "Group",
                       data = dat,
                       factorVars = vars_cat,
                       addOverall = TRUE)

print(tab1, showAllLevels = TRUE, quote = FALSE, noSpaces = TRUE)

# Optional: export Table 1 to CSV
# write.csv(print(tab1, quote = FALSE, noSpaces = TRUE), "Table1_Baseline.csv")


# ---- 3) Logistic regression (Table 2) ----
# Univariate logistic regression for candidate predictors
candidate_vars <- c("Age","BMI","FIGO_Stage","Histology","PDL1_Positive",
```

```
                    "Prior_Chemotherapy","Prior_Radiotherapy",
                    "NLR","LDH_U_L","Hemoglobin_g_L","Albumin_g_L",
                    "CEA_ng_mL","CA125_U_mL")

# Helper function
fit_uni <- function(v){
  f <- as.formula(paste0("PD1_Resistance ~ ", v))
  m <- glm(f, data = dat, family = binomial)
  tidy(m, exponentiate = TRUE, conf.int = TRUE) %>%
    filter(term != "(Intercept)") %>%
    mutate(variable = v) %>%
    select(variable, term, estimate, conf.low, conf.high, p.value)
}

uni_res <- map_dfr(candidate_vars, fit_uni)

# For LDH per 10 U/L increase (as in manuscript)
m_ldh10 <- glm(PD1_Resistance ~ I(LDH_U_L/10), data = dat, family = binomial)
ldh10_out <- tidy(m_ldh10, exponentiate = TRUE, conf.int = TRUE) %>%
  filter(term != "(Intercept)") %>%
  transmute(variable = "LDH (per 10 U/L)",
            term,
            OR = estimate,
            CI_low = conf.low,
            CI_high = conf.high,
            p = p.value)

# Multivariable model: NLR + LDH(per 10) + PD-L1 + prior chemotherapy
m_multi <- glm(PD1_Resistance ~ NLR + I(LDH_U_L/10) + PDL1_Positive + Prior_Chemotherapy,
               data = dat, family = binomial)

multi_out <- tidy(m_multi, exponentiate = TRUE, conf.int = TRUE) %>%
  filter(term != "(Intercept)") %>%
  transmute(term,
            OR = estimate,
            CI_low = conf.low,
            CI_high = conf.high,
            p = p.value)

# Model fit check (optional)
# Hosmer-Lemeshow goodness-of-fit (grouped by deciles)
hoslem.test(dat$PD1_Resistance, fitted(m_multi), g = 10)

# ---- 4) ROC analysis + DeLong tests (Table 3 / Figure 1) ----
# Predicted probabilities for combined model
dat$pred_combined <- predict(m_multi, type = "response")

roc_nlr <- roc(dat$PD1_Resistance, dat$NLR, quiet = TRUE)
roc_ldh <- roc(dat$PD1_Resistance, dat$LDH_U_L, quiet = TRUE)
roc_pdl1 <- roc(dat$PD1_Resistance, as.numeric(dat$PDL1_Positive) - 1, quiet = TRUE)  # 0/1
roc_chemo <- roc(dat$PD1_Resistance, as.numeric(dat$Prior_Chemotherapy) - 1, quiet = TRUE)  # 0/1
roc_comb <- roc(dat$PD1_Resistance, dat$pred_combined, quiet = TRUE)

# AUC + 95% CI
auc_ci <- function(r){
  c(AUC = as.numeric(auc(r)),
    CI_low = as.numeric(ci.auc(r)[1]),
    CI_high = as.numeric(ci.auc(r)[3]))
}

auc_tbl <- rbind(
  NLR = auc_ci(roc_nlr),
  LDH = auc_ci(roc_ldh),
  PDL1 = auc_ci(roc_pdl1),
  Chemo = auc_ci(roc_chemo),
  Combined = auc_ci(roc_comb)
```

```
) %>% as.data.frame()

print(auc_tbl)

# Optimal cut-offs using Youden index (continuous markers)
# pROC has coords() for thresholds
nlr_cut <- coords(roc_nlr, "best", best.method = "youden",
                  ret = c("threshold","sensitivity","specificity"), transpose = FALSE)

ldh_cut <- coords(roc_ldh, "best", best.method = "youden",
                  ret = c("threshold","sensitivity","specificity"), transpose = FALSE)

comb_cut <- coords(roc_comb, "best", best.method = "youden",
                   ret = c("threshold","sensitivity","specificity"), transpose = FALSE)

print(nlr_cut); print(ldh_cut); print(comb_cut)

# DeLong tests: compare combined vs each single predictor
delong_nlr  <- roc.test(roc_comb, roc_nlr, method = "delong")
delong_ldh  <- roc.test(roc_comb, roc_ldh, method = "delong")
delong_pdl1 <- roc.test(roc_comb, roc_pdl1, method = "delong")
delong_chemo<- roc.test(roc_comb, roc_chemo, method = "delong")

delong_results <- tibble(
  Comparison = c("Combined vs NLR","Combined vs LDH","Combined vs PD-L1","Combined vs Prior chemo")
  p_value = c(delong_nlr$p.value, delong_ldh$p.value, delong_pdl1$p.value, delong_chemo$p.value)
)

print(delong_results)

# Plot ROC curves (Figure 1)
pdf("Figure1_ROC.pdf", width = 6.5, height = 6)
plot(roc_nlr, print.auc = TRUE, main = "ROC Curves for PD-1 Resistance")
plot(roc_ldh, add = TRUE)
plot(roc_comb, add = TRUE, lwd = 2)
legend("bottomright",
       legend = c("NLR","LDH","Combined"),
       lwd = c(1,1,2),
       bty = "n")
dev.off()


# ---- 5) Sensitivity analyses (examples) ----
# Example A: Alternative NLR thresholds (3.0–3.8) and LDH thresholds (230–250)
alt_nlr <- c(3.0, 3.2, 3.4, 3.6, 3.8)
alt_ldh <- c(230, 240, 250)

sens_tbl <- expand.grid(NLR_cut = alt_nlr, LDH_cut = alt_ldh) %>%
  as_tibble() %>%
  mutate(
    NLR_high = ifelse(dat$NLR >= NLR_cut, 1, 0) %>% list(),
    LDH_high = ifelse(dat$LDH_U_L >= LDH_cut, 1, 0) %>% list()
  )

# Practical approach: loop to compute AUC of a simplified binary score
calc_auc_binary_score <- function(nlr_cut, ldh_cut){
  score <- (dat$NLR >= nlr_cut) + (dat$LDH_U_L >= ldh_cut) +
           (as.numeric(dat$PDL1_Positive) - 1 == 0) +  # PD-L1 negative as risk
           (as.numeric(dat$Prior_Chemotherapy) - 1 == 1)
  r <- roc(dat$PD1_Resistance, score, quiet = TRUE)
  as.numeric(auc(r))
}

sens_out <- expand.grid(NLR_cut = alt_nlr, LDH_cut = alt_ldh) %>%
  as_tibble() %>%
  rowwise() %>%
```

```
  mutate(AUC = calc_auc_binary_score(NLR_cut, LDH_cut)) %>%
  ungroup()

print(sens_out)

# Example B: Leave-one-out (LOO) AUC for combined model (computationally heavier)
# LOO on logistic regression predicted probabilities
loo_auc <- function(){
  preds <- rep(NA, nrow(dat))
  for(i in 1:nrow(dat)){
    m <- glm(PD1_Resistance ~ NLR + I(LDH_U_L/10) + PDL1_Positive + Prior_Chemotherapy,
             data = dat[-i,], family = binomial)
    preds[i] <- predict(m, newdata = dat[i,], type = "response")
  }
  roc(dat$PD1_Resistance, preds, quiet = TRUE) %>% auc() %>% as.numeric()
}
# loo_auc_value <- loo_auc()
# print(loo_auc_value)


# ---- 6) Subgroup analyses (examples) ----
# Example: by FIGO stage
for(st in levels(dat$FIGO_Stage)){
  sub <- dat %>% filter(FIGO_Stage == st)
  m <- glm(PD1_Resistance ~ NLR + I(LDH_U_L/10) + PDL1_Positive + Prior_Chemotherapy,
           data = sub, family = binomial)
  sub$pred <- predict(m, type = "response")
  r <- roc(sub$PD1_Resistance, sub$pred, quiet = TRUE)
  cat("\nFIGO Stage", st, ": AUC =", as.numeric(auc(r)), "\n")
}

# ---- 7) Table 4: Combination therapy outcomes (manual entry) ----
# If you have patient-level outcomes for the 18 resistant patients, merge here.
# Otherwise, you can report Table 4 as summarized counts, as in the manuscript.

# End of script
```
