# Supplementary material for: Baseline inflammatory and metabolic indicators associated with early PD-1 inhibitor resistance in advanced cervical cancer: a retrospective cohort study
Source: Front Med (Lausanne). 2026 Mar 16;13:1779898. doi: 10.3389/fmed.2026.1779898 (PMC13033690; doi:10.3389/fmed.2026.1779898)
Supplement: Supplementary file 3 [file Table_1.docx]

**Table S1. Sensitivity Analyses of the Predictive Model for PD-1 Inhibitor Resistance**

| **Analysis condition** | **NLR cut-off** | **LDH cut-off (U/L)** | **n (included)** | **AUC (95% CI)** | **Sensitivity (%)** | **Specificity (%)** | **Youden Index** | **P value (vs primary)** |
| --- | --- | --- | --- | --- | --- | --- | --- | --- |
| **Primary model** (NLR = 3.45; LDH = 238.5) | 3.45 | 238.5 | 140 | **0.842 (0.773–0.911)** | **80.6** | **76.4** | **0.57** | — |
| Alternative #1 (lower cut-offs) | 3.00 | 230.0 | 140 | 0.837 (0.764–0.901) | 82.4 | 73.6 | 0.56 | 0.642 |
| Alternative #2 (higher cut-offs) | 3.80 | 250.0 | 140 | 0.846 (0.773–0.912) | 78.3 | 78.5 | 0.57 | 0.713 |
| Excluding patients with missing labs (n = 8) | 3.45 | 238.5 | 132 | 0.839 (0.766–0.901) | 80.3 | 76.0 | 0.56 | 0.711 |
| Excluding NLR > 10.0 (outliers) | 3.45 | 238.5 | 137 | 0.841 (0.771–0.905) | 81.0 | 75.6 | 0.57 | 0.851 |
| Leave-one-out validation (LOOCV) | — | — | 140 | 0.839 (0.770–0.902) | — | — | — | 0.929 |

* No statistically significant difference was observed between any sensitivity scenario and the primary model (all P > 0.05).

**Table S2. Subgroup Analyses of the Combined Predictive Model for PD-1 Inhibitor Resistance**

| **Subgroup variable** | **Category** | **n** | **AUC (95% CI)** | **Sensitivity (%)** | **Specificity (%)** | **Youden Index** | **P value (vs reference)** |
| --- | --- | --- | --- | --- | --- | --- | --- |
| **Treatment regimen** |  |  |  |  |  |  |  |
| PD-1 monotherapy | 64 | 0.841 (0.746–0.917) | 78.8 | 76.7 | 0.56 | — |  |
| PD-1 combination therapy (chemo ± anti-VEGF)** | 76 | 0.835 (0.742–0.912) | 82.4 | 74.6 | 0.57 | 0.628 |  |
| **PD-L1 expression** |  |  |  |  |  |  |  |
| High expression (≥1%) | 83 | 0.846 (0.768–0.918) | 79.5 | 77.3 | 0.57 | — |  |
| Low expression (<1%) | 57 | 0.831 (0.731–0.909) | 81.1 | 75.0 | 0.56 | 0.714 |  |
| **FIGO stage** |  |  |  |  |  |  |  |
| Stage III–IVa | 92 | 0.837 (0.752–0.907) | 79.8 | 76.1 | 0.56 | — |  |
| Stage IVb | 48 | 0.848 (0.759–0.919) | 81.3 | 77.5 | 0.58 | 0.672 |  |

* AUCs were compared across subgroups using DeLong’s test.
** Combination therapy includes PD-1 plus chemotherapy and/or anti-angiogenic agents.
All P > 0.05 indicate no significant difference between subgroups.
